# A randomized controlled trial of a proportionate universal parenting program delivery model (E-SEE Steps) to enhance child social-emotional wellbeing

**DOI:** 10.1371/journal.pone.0265200

**Published:** 2022-04-04

**Authors:** Tracey Bywater, Vashti Berry, Sarah Blower, Matthew Bursnall, Edward Cox, Amanda Mason-Jones, Sinead McGilloway, Kirsty McKendrick, Siobhan Mitchell, Kate Pickett, Gerry Richardson, Kiera Solaiman, M. Dawn Teare, Simon Walker, Karen Whittaker

**Affiliations:** 1 Department of Health Sciences, University of York, York, North Yorkshire, United Kingdom; 2 College of Medicine and Health, University of Exeter, Exeter, Devon, United Kingdom; 3 Sheffield Clinical Trials Research Unit, Sheffield, South Yorkshire, United Kingdom; 4 Centre for Health Economics, University of York, York, North Yorkshire, United Kingdom; 5 Centre for Mental Health and Community Research, Maynooth University, Maynooth, Co Kildare, Ireland; 6 Institute of Health and Society University, Newcastle upon Tyne, Tyne and Wear, United Kingdom; 7 School of Nursing, University of Central Lancashire, Preston, Lancashire, United Kingdom; Illawarra Shoalhaven Local Health District, AUSTRALIA

## Abstract

**Background:**

Evidence for parenting programs to improve wellbeing in children under three is inconclusive. We investigated the fidelity, impact, and cost-effectiveness of two parenting programs delivered within a longitudinal proportionate delivery model (‘E-SEE Steps’).

**Methods:**

Eligible parents with a child ≤ 8 weeks were recruited into a parallel two-arm, assessor blinded, randomized controlled, community-based, trial with embedded economic and process evaluations. Post-baseline randomization applied a 5:1 (intervention-to-control) ratio, stratified by primary (child social-emotional wellbeing (ASQ:SE-2)) and key secondary (maternal depression (PHQ-9)) outcome scores, sex, and site. All intervention parents received the Incredible Years^®^ Baby Book (IY-B), and were offered the targeted Infant (IY-I)/Toddler (IY-T) program if eligible, based on ASQ:SE-2/PHQ-9 scores. Control families received usual services. Fidelity data were analysed descriptively. Primary analysis applied intention to treat. Effectiveness analysis fitted a marginal model to outcome scores. Cost-effectiveness analysis involved Incremental Cost-Effectiveness Ratios (ICERs).

**Results:**

The target sample (N = 606) was not achieved; 341 mothers were randomized (285:56), 322 (94%) were retained to study end. Of those eligible for the IY-I (n = 101), and IY-T (n = 101) programs, 51 and 21 respectively, attended. Eight (of 14) groups met the 80% self-reported fidelity criteria. No significant differences between arms were found for adjusted mean difference scores; ASQ:SE-2 (3.02, 95% CI: -0.03, 6.08, p = 0.052), PHQ-9 (-0.61; 95% CI: -1.34, 0.12, p = 0.1). E-SEE Steps had higher costs, but improved mothers’ Health-related Quality of Life (0.031 Quality Adjusted Life Year (QALY) gain), ICER of £20,062 per QALY compared to control. Serious adverse events (n = 86) were unrelated to the intervention.

**Conclusions:**

E-SEE Steps was not effective, but was borderline cost-effective. The model was delivered with varying fidelity, with lower-than-expected IY-T uptake. Changes to delivery systems and the individual programs may be needed prior to future evaluation.

**Trial registration:**

International Standard Randomized Controlled Trial Number: ISRCTN11079129.

## Introduction

Behavioral and mental disorders have become a public health crisis [1]. Early intervention/prevention may prevent child mental health issues, and mitigate personal, familial, and societal costs of later life negative outcomes [2]. Evidence-based group parent programs are effective for parents with children aged three years or older in reducing/preventing child conduct problems and increasing social-emotional wellbeing [3]. Program evidence is lacking for parents of children under two [2].

The Incredible Years^®^ (IY) manualized parent programs (www.incredibleyears.com) aim to enhance child wellbeing for children aged 0–12 years. IY has a solid evidence base for parents of children aged three upwards [4], and families with severe depression and severe conduct problems demonstrate positive co-occurring changes following attendance [5]. Parental depression can lead to unresponsive/ineffective parenting strategies and (inadvertent) emotional neglect [6]. In the UK IY Infant (IY-I) program has shown promise of effectiveness in a comparison study [7] although IY Toddler (IY-T) program effectiveness remains inconclusive [8]. More research is needed on these programs to establish if they are effective when delivered as ‘standalone’ interventions [4], or in a longitudinal model as, and when, parents may need support as their children grow.

Trials of “standalone” interventions do not consider cumulative doses of one or more interventions for families with differing needs at different times. Proportionate universal approaches may reduce health inequality by offering support/services to meet individual/family need in preventative and/or treatment delivery [9]. Such approaches, although under-utilized, appear useful for mental health interventions [10].

This study tested a proportionate, longitudinal, universal intervention model called “**E**nhancing **S**ocial-**E**motional Health and Wellbeing in the **E**arly Years (E-SEE) Steps”—comprising a universal step (Incredible Babies Book; IY-B) plus targeted IY-I and IY-T steps. The study was conducted in response to a 2013 funding call from the (UK) National Institute for Health Research (NIHR), Public Health Research (PHR) to address the evidence gap around the effectiveness of parent programs in enhancing wellbeing in children under two. We chose IY because of the program’s existing evidence base for children aged 3+, and its suite of age-appropriate programs (infant, toddler, etc. up to age 12 years) which are well suited to a proportionate, longitudinal, universal delivery model. The objectives of the study were to assess if ‘E-SEE Steps’ can:

- Enhance child social-emotional wellbeing at 20 months of age when compared with services as usual,
- be delivered with fidelity as a proportionate, longitudinal, universal model,
- be cost-effective at 20 months when compared with services as usual.

## Materials and methods

### Study design

The study involved a multi-center pragmatic parallel two-arm, assessor blinded, randomized controlled trial (RCT) with embedded process and economic evaluations, within community settings in England. Recruitment began in May 2017 and data were collected at home visits by trained data collectors at baseline, follow-up 1 (FU1) (2 months post baseline), follow-up 2 (FU2) (9 months post baseline) and follow-up 3 (FU3) (18 months post-baseline). Follow-ups were completed in March 2020. We evaluated the overall effect of IY (delivered in the context of E-SEE Steps) on child social-emotional wellbeing and parent depression at 20 months of age. A pilot study [11] (N = 205) informed the trial leading to amendments (e.g. changes to sample size and random allocation ratio) which can be found in the full protocol (see https://www.dev.fundingawards.nihr.ac.uk/award/13/93/10) and published protocol [12]. We followed CONSORT (see S1 Table), CHEERS and TIDieR reporting guidance.

### Participants and settings

Eligible parents with a child ≤ 8 weeks were recruited from community settings across four local authorities in England (two North, one Mid and one in the South). Parents had to be willing to be randomized, able to receive the intervention and to provide written informed consent. Parents were excluded if they were enrolled on another group-based program or had a child with obvious/diagnosed organic child developmental difficulties.

Health visitors and family services asked eligible families if they would like information on the study. Those who agreed were contacted, with consent, by the research team. Researchers obtained written informed consent during home visits in accordance with ethical guidance and approvals. Parents could also self-refer and invite co-parents to participate. Families received a shopping voucher at each data collection point as a small ‘thank you’ for their contributions (increasing in £5 increments at each time-point, from £15-£30).

### Measures

Measure selection was informed by systematic reviews [13, 14] and Parenting Advisory Committee (PAC) feedback (see **Full Protocol** at https://www.dev.fundingawards.nihr.ac.uk/award/13/93/10 for detailed information on measures). Demographic data included: age, ethnicity, religion, income, marital status, parent education, housing, family composition, infant feeding, prematurity.

Child social-emotional wellbeing was the primary outcome, assessed by the Ages and Stages Questionnaire: Social-emotional, Second Edition (ASQ:SE-2) [15]. The ASQ:SE-2 has several age-appropriate versions with different scoring, all of which include ‘low/no risk’ ‘monitoring zone’ and ‘refer zone’ ranges. Test-retest reliability is 89%, internal consistency is 84%, sensitivity is 81%, and specificity is 84%. The minimum clinically significant difference for the trial was defined as 5 points. Parent depression was the key secondary outcome, assessed by the widely used and psychometrically robust 9-item Patient Health Questionnaire (PHQ-9) [16]. Categories include ‘no’ (< = 4), ‘mild’ (5–10, ‘moderate’ (10–14); ‘moderately severe’ (15–19), and ‘severe’ (20–27) depression. The ASQ:SE-2 and the PHQ-9 were administered at all timepoints.

Other secondary outcome measures included The Parent Sense of Competence (PSOC) [17] which assesses parent satisfaction and efficacy, and the CARE Index (Infancy) [18], which is an observational measure of parent-child relationships. Both were administered at all timepoints. The Strengths and Difficulties Questionnaire (SDQ: 2-4-yr Version) [19] which assesses child behavior and emotions, and the Maternal Postnatal Attachment Scale (MPAS) [20] which assesses maternal bonding were administered at the final timepoint only.

### Sample size calculation

Sample size was calculated using the ASQ:SE-2, and the values for key design parameters were informed by, and estimated from, the pilot study [11]; for further information see the published protocol [12] and full protocol (https://www.dev.fundingawards.nihr.ac.uk/award/13/93/10). The clinically important difference at FU3 (18 months post-baseline) was defined as 5 units of the ASQ:SE-2. We expected a consistent effect over the three follow-ups, with an assumed SD of 18 on the ASQ:SE-2 at FU3. The correlation between baseline and FU3 was 0.26, and between pairs of measurements after baseline, was 0.4. Due to the group-based nature of the intervention, a design effect of 1.25 was applied as an inflation factor for the intervention arm. We required two-sided 5% significance level and 90% power. A 5:1 randomization ratio, intervention to control, was necessary to ensure that sufficient parents would meet the proportionate criteria to attend the parent programs, with a viable group size. A target of N = 606 allowed for 12% attrition; 441 intervention and 92 control parents needed to be retained.

### Randomization and blinding

Randomization was conducted post-baseline by EpiGenysis at the University of Sheffield, using a web-based system with a 5:1 (intervention to control) ratio. Stratification variables included baseline PHQ-9, child ASQ:SE-2 scores, child and parent sex, and research site. All fieldworkers, referral agents, the chief investigator, statisticians (until final analysis), and the Trial Steering Committee, were blind to allocation. Participants, IY leaders, trial and data managers, and the process evaluation team, were not blind.

### Intervention

E-SEE Steps comprises two IY programs (IY-I and IY-T) delivered in a proportionate, longitudinal, universal intervention model with three steps—one universal, and two subsequent targeted/indicated steps, as the children age (S1 Fig). The IY-B was posted to all intervention parents to increase awareness of their babies’ socioemotional needs. The IY-I and IY-T targeted group sessions were delivered weekly in collaborative two-hour sessions which include video clips of real-life situations and group discussions, plus exercises to practice at home. IY is underpinned by both social learning and attachment theory [21, 22]. Program content is summarized in S2 Table.

E-SEE-Steps was delivered by Early Years Children’s Services and/or Public Health Nursing staff, who were trained by accredited IY mentors (and supervised regularly) to deliver IY as part of the trial.

Parents were eligible for the IY-I or IY-T programs if they were obtained ‘mildly depressed’ or higher scores on the PHQ-9, or if their child scored in the ‘monitoring zone or above’ on the ASQ:SE-2 (suggesting potential social-emotional issues) at follow-up 1 or 2. The research team contacted parents, if eligible for IY-I/T, and sites engaged with parents in relation to program attendance. There were four possible intervention ‘doses’ that the trial sample could have, dependent on their level of need: IY-B only; IY-B **plus** IY-I; IY-B **plus** IY-I **and** IY-T; or IY-B **plus** IY-T (for IY-I and IY-T logic models see http://www.incredibleyears.com/about/incredible-years-series/series-goals/). The control group/arm received services as usual (SAU) which included a range of supports, including behavior management, healthy weight/nutrition, early learning and development, and post-natal support. IY-B, IY-I and IY-T were not offered as SAU in trial delivery sites.

### Process evaluation

Fidelity monitoring data included receipt of the IY-B, and IY-I and IY-T group attendance and parent satisfaction (using standard IY weekly feedback forms), leader self-rated adherence using IY weekly checklists, and researcher-rated implementation fidelity using the Parent Programme Implementation Checklist (PPIC) [23]. The PPIC measures adherence, quality of delivery and participant responsiveness. Barriers and facilitators to delivery, and stakeholder experiences, are reported separately [24].

### Economic evaluation

The cost-effectiveness evaluation utilized data from an adapted Client Services Receipt Inventory (CSRI) [25] which assessed parent and child access to health, social and community services. The SDQ [19], which measures child behavior and emotions, the Pediatric Quality of Life Inventory (PEDsQL) [26], and EQ-5D5L [27], which assesses adult health dimensions of mobility, self-care, usual activities, pain/discomfort, and anxiety/depression were used to calculate Quality Adjusted Life Years (QALYs) [28].

### Ethical considerations

The study was approved by the National Health Service (NHS) North Wales Research Ethics Committee (REC) 5, Bangor on 22nd May 2015 (REC Reference: 15/WA/0178, IRAS 173946), and by Departmental Ethics Committee, University of York on 10th August 2015 (Reference: FC15/03). All participants provided written informed consent.

### Analysis

For the effectiveness analysis, a marginal model was fitted to the ASQ:SE-scores of children when approximately 4, 11 and 20-months old (FUs 1–3), using general estimating equations with a Gaussian family, identity link, robust standard errors and autoregressive covariance structure of order 1 AR(1). STATA/MP 16.0 was used, with a two-sided test at the 5% level. Primary analysis applied intention to treat.

Baseline prognostic factors, potential confounding factors, follow-up time and delivery site were included as covariates. Sensitivity analyses assessed the robustness of the primary analysis using the standard techniques in the RCT literature. For example, item non-response was imputed using questionnaire developer rules, and missing outcomes were explored by Multiple Imputation using Chained Equations (MICE) [29]. For further details see our Statistical Analysis Plan (SAP) at https://www.york.ac.uk/media/healthsciences/documents/research/public-health/e-see/1_Statistical%20Analysis%20Plan%20(main%20trial).pdf Prior to database close and un-blinding four changes were agreed and made to the analysis model.

1. An original multilevel mixed model with treatment group and participants as random effects was replaced with a marginal model fitted using GEE. We no longer accounted for treatment group clustering because the offer of IY-I and IY-T was conditional on FU1 and FU2 outcomes, so clustering was confounded with treatment effect. We used a marginal model because accounting for repeated measures using a mixed model, inflates the Type 1 error, or gives a biased estimate of the treatment effect. Simulations conducted during SAP development, suggested estimates from this alternative model were robust to Inter-Cluster Correlations (ICCs) below 0.2.
2. Cluster-level analysis using summary measures is no longer included because participants can get IY-I alone, IY-T alone or both, so there is no way of grouping participants into clusters that remain stable throughout the intervention.
3. The sex of primary caregiver covariate is not used because findings from the pilot showed no male primary caregivers for the associated model parameter to be estimated.
4. Per protocol and Complier Average Causal Effect (CACE) analysis were not conducted as there is no satisfactory way of defining compliers without biasing the estimated impact of IT-I and IY-T on compliers. This is due to the conditional design whereby eligible participants have already scored highly on the outcome measure. Descriptive analysis of the characteristics associated with compliance was undertaken.

Fidelity monitoring data were analyzed descriptively using means/medians and percentages.

Cost-effectiveness was assessed using incremental cost per QALY [28] gained of E-SEE steps compared with SAU. Analyses were conducted with probabilistic sensitivity analyses used to estimate the uncertainty around the adoption decision. Sensitivity analyses determined the robustness of the results to altering leading assumptions, see S1 Text.

Costs were estimated from a public sector perspective and calculated by applying published national (UK) cost estimates to relevant resource use. Costs and effects were discounted at 3.5% per annum as per national guidance from the National Institute of Health and Care Excellence (NICE) [30]. Outcomes were assessed in terms of QALYs [28], using SDQ [19] mapped to PEDsQL for children [26], and EQ-5D5L for adults [27].

## Results

**Fig 1:**
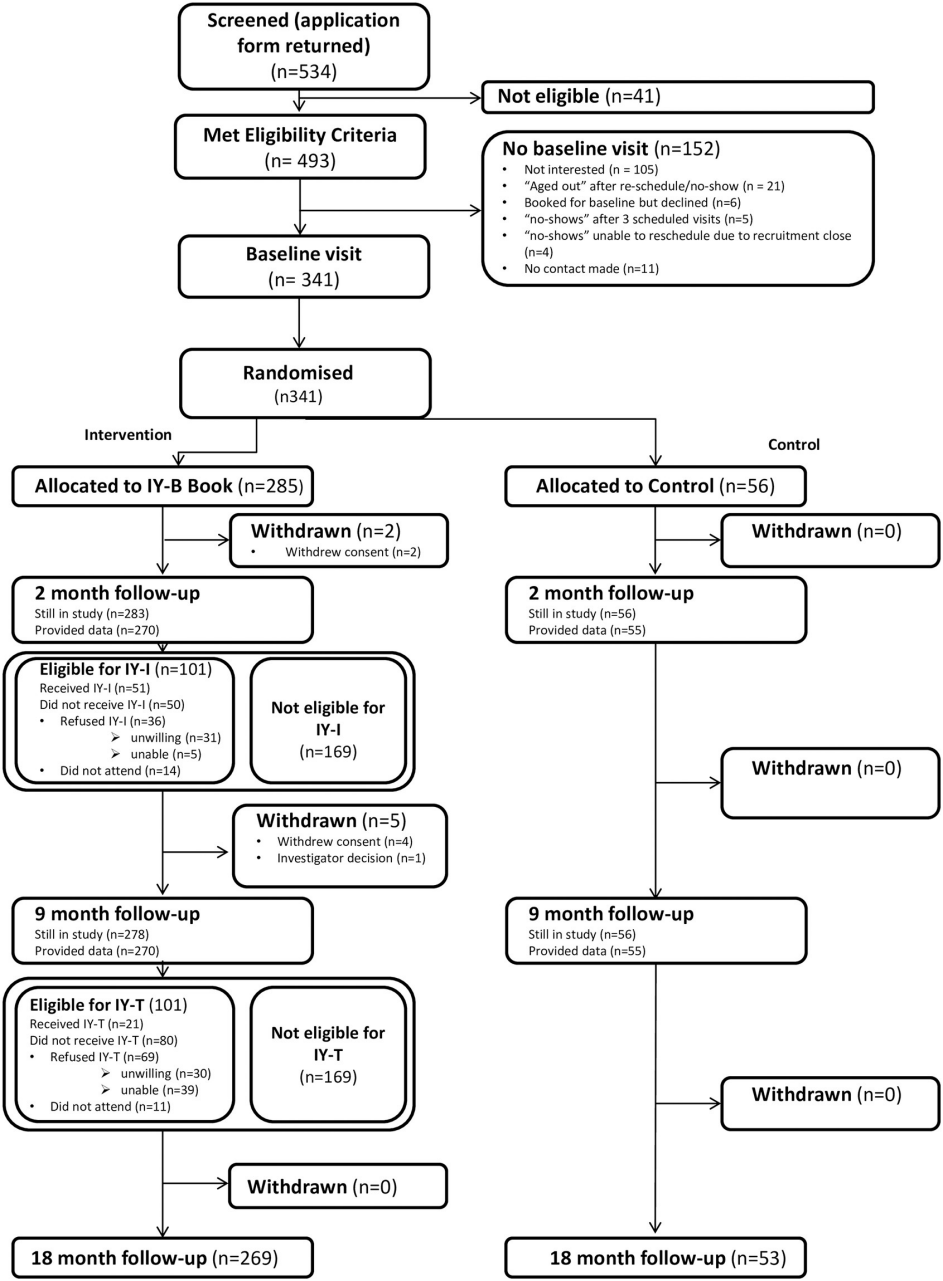
Consort flow chart.

A total of 341 eligible mothers (from a potential 493) were randomized (see Fig 1) and their data analyzed; 322 (94%) were retained at trial end (6 withdrew, and 1 was withdrawn by the CI). The target sample size of 606 was not achieved. Mothers’ mean age was 30.9 (5.0) years, mean child age was 6 (2.1) weeks (see Table 1). No major imbalances between arms at baseline existed in terms of covariates and baseline outcome scores.

**Table 1:** Baseline demographic characteristics of child and primary caregiver.

		Intervention	Control	All
**Child**		(n = 285)	(n = 56)	(n = 341)
**Categorical Variables**				
**Sex of Child**				
	Male	145 (51%)	29 (52%)	174 (51%)
	Female	140 (49%)	27 (48%)	167 (49%)
**Child’s Ethnicity**				
	English/Welsh/Scottish/Northern Irish/British	215 (75%)	37 (66%)	252 (74%)
	Any other White background	9 (3%)	4 (7%)	13 (4%)
	White and Black Caribbean	2 (1%)	1 (2%)	3 (1%)
	White and Black African	3 (1%)	1 (2%)	4 (1%)
	White and Asian	5 (2%)	0 (0%)	5 (1%)
	Any Other Mixed/Multiple ethnic group	6 (2%)	0 (0%)	6 (2%)
	Indian	14 (5%)	6 (11%)	20 (6%)
	Pakistani	19 (7%)	5 (9%)	24 (7%)
	Bangladeshi	4 (1%)	0 (0%)	4 (1%)
	Any other Asian background	2 (1%)	0 (0%)	2 (1%)
	African	5 (2%)	1 (2%)	6 (2%)
	Any other ethnic group please describe	1 (0%)	1 (2%)	2 (1%)
**Premature**				
	No	274 (96%)	53 (95%)	327 (96%)
	Yes	9 (3%)	3 (5%)	12 (4%)
	Missing Data	2 (1%)	0 (0%)	2 (1%)
**Difficulties at birth**				
	No	132 (46%)	26 (46%)	158 (46%)
	Yes	153 (54%)	30 (54%)	183 (54%)
**Continuous variables**				
**Child’s age (weeks)**				
	N (%)	285 (100%)	56 (100%)	341 (100%)
	Mean (SD)	6.1 (2.1)	5.9 (2.2)	6.0 (2.1)
	Median (IQR)	6 (4, 8)	6 (4, 8)	6 (4, 8)
	Min., Max.	2, 11	2, 10	2, 11
**Primary caregiver**		(n = 285)	(n = 56)	(n = 341)
**Categorical Variables**				
**Parent’s age group**				
	18 to 21	9 (3%)	2 (4%)	11 (3%)
	22 to 25	36 (13%)	7 (13%)	43 (13%)
	26 to 30	88 (31%)	15 (27%)	103 (30%)
	31 to 35	95 (33%)	21 (38%)	116 (34%)
	36 and above	57 (20%)	11 (20%)	68 (20%)
**Sex**				
	Female	285 (100%)	56 (100%)	341 (100%)
**Ethnicity**				
	English/Welsh/Scottish/Northern Irish/British	218 (76%)	38 (68%)	256 (75%)
	Any other White background	14 (5%)	4 (7%)	18 (6%)
	Mixed/Multiple ethnic group	6 (2%)	2 (4%)	8 (2%)
	Indian	15 (5%)	7 (13%)	22 (6%)
	Pakistani	18 (6%)	3 (5%)	21 (6%)
	Any other Asian background	7 (3%)	0 (0%)	7 (2%)
	Any other ethnic group	7 (3%)	2 (4%)	9 (3%)
**Highest qualification previously achieved**				
	Post doctorate	8 (3%)	0 (0%)	8 (2%)
	Masters’ degree	28 (10%)	8 (14%)	36 (11%)
	Undergraduate degree e.g. BA or BSc	96 (34%)	14 (25%)	110 (32%)
	A certificate or diploma in higher education	33 (12%)	5 (9%)	38 (11%)
	A, AS or S levels	19 (7%)	7 (13%)	26 (8%)
	O levels or GCSE: 5 or more	15 (5%)	6 (11%)	21 (6%)
	O levels or GCSE: 4 or less	9 (3%)	3 (5%)	12 (4%)
	Overseas qualifications	10 (4%)	2 (4%)	12 (4%)
	Vocational qualifications	53 (19%)	8 (14%)	61 (18%)
	None of these qualifications	14 (5%)	1 (2%)	15 (4%)
	Missing Data	0 (0%)	2 (4%)	2 (1%)
**Relationship status**				
	Married and living together	184 (65%)	38 (68%)	222 (65%)
	Cohabiting/living together	70 (25%)	12 (21%)	82 (24%)
	Living together part of the time	4 (1%)	3 (5%)	7 (2%)
	Separated	4 (1%)	0 (0%)	4 (1%)
	A couple but not living together	13 (5%)	0 (0%)	13 (4%)
	Dating	1 (0%)	1 (2%)	2 (1%)
	Not in a relationship	9 (3%)	2 (4%)	11 (3%)
**Continuous variables**				
**Age**				
	N (%)	285 (100%)	56 (100%)	341 (100%)
	Mean (SD)	30.9 (5.1)	31.1 (5.0)	30.9 (5.0)
	Median (IQR)	31 (28, 35)	32 (27, 34)	31 (28, 34)
	Min., Max.	18, 43	20, 40	18, 43
**Baseline weekly Income**				
	N (%)	226 (79%)	43 (77%)	269 (79%)
	Mean (SD)	733.1 (470.7	766.9 (454.4	738.5 (467.5
	Median (IQR)	603 (400, 95	710 (400, 10	630 (400, 97
	Min., Max.	0, 2500	151, 1850	0, 2500

### Process evaluation—Fidelity, intervention take-up and delivery

The (universal) IY-B was posted to all intervention families. Fifty-one from 101 eligible at FU1 received at least one session of IY-I, and 21 from 101 eligible at FU2 received at least one IY-T session (see Fig 1 and S3 Table). We expected uptake to be 50 and 48 respectively, showing a lower-than-expected IY-T uptake. Attendance levels reduced over time; 80% attended the fifth session, which reduced to 45% for IY-I at session 8 (of 9) and 43% at session 10 (of 12) for IY-T. Average individual session attendance was 6.5 and 6.4 for IY-I and IY-T respectively.

Parents who attended at least one IY-I/IY-T session and parents who were invited but did not attend did not differ on outcomes, although better educated parents in higher income bands were marginally more likely to take up the intervention; numbers were small and therefore limit any definitive conclusions, see S4 Table. In addition, we compared participants eligible for IY-I and IY-T with subgroups of control participants with eligible ASQ:SE-2 and PHQ-9 scores (pseudo controls), using the same model as for the primary outcome. No differences were found between arms, see S5 Table). Parents with lower depression (PHQ-9) scores were more likely to attend at least one IY-I session. There was no difference in attendance by ASQ:SE-2 scores.

Weekly parent satisfaction with content and process was high, averaging 3.4 and 3.7 (out of 4), for IY-I and IY-T respectively.

Six (of eight) IY-I and two (of six) IY-T groups met the trial-set criteria of 80% on self-reported fidelity (adherence). The independent PPIC observation [23] yielded average fidelity rates across quality, adherence, and responsiveness of 64% and 74% for IY-I and IY-T respectively (S2 Fig).

### Effectiveness evaluation

The findings show that ESEE-Steps was not effective in enhancing child social-emotional wellbeing compared to the control arm. Primary analyses found a borderline statistically significant difference in favor of the control arm (3.02, 95%CI: -0.03, 6.08, p = 0.052) (Table 2). On average ASQ:SE-2 scores tended to be 3 units higher over the three FUs in the E-SEE steps arm when compared to controls. Unplanned sensitivity analyses were performed due to skewed data and the implication of this with a large arm size imbalance, due to the randomization ratio of 5:1. Sensitivity analysis provided no evidence that the Minimal Clinically Important Difference (MCID) was reached, see S6 Table. The difference between groups was reduced for the ASQ:SE2, but did not change the primary analysis (2.56, 95%CI: -0.69, 5.80, p = 0.122). The results did not differ depending on parent education, child sex or if their child was first-born.

**Table 2:** Difference between arms for primary and key secondary outcomes.

		Intervention	Control	All	Differences		p-value
		(n = 285)	(n = 56)	(n = 341)	Mean diff (95% CI)	Adjusted Mean diff (95% CI)	
**ASQ:SE2 BL**	N (%)	285 (100%)	56 (100%)	341 (100%)			
	Mean (SD)	22.8 (15.1)	23.8 (16.6)	23.0 (15.4)	-0.91 (-5.32, 3.50)		
	Median (IQR)	20 (10, 30)	20 (15, 30)	20 (15, 30)			
	Min., Max.	0, 105	0, 95	0, 105			
**FU1**	N (%)	270 (95%)	55 (98%)	325 (95%)			
	Mean (SD)	20.5 (15.7)	16.5 (10.9)	19.8 (15.1)	4.06 (-0.29, 8.41)		
	Median (IQR)	15 (10, 30)	15 (5, 20)	15 (10, 25)			
	Min., Max.	0, 105	0, 50	0, 105			
**FU2**	N (%)	269 (94%)	55 (98%)	324 (95%)			
	Mean (SD)	29.0 (16.2)	26.8 (14.7)	28.6 (16.0)	2.14 (-2.50, 6.78)		
	Median (IQR)	25 (20, 40)	30 (15, 40)	25 (20, 40)			
	Min., Max.	0, 100	0, 65	0, 100			
**FU3**	N (%)	268 (94%)	53 (95%)	321 (94%)			
	Mean (SD)	26.8 (19.5)	28.1 (23.6)	27.0 (20.2)	-1.30 (-7.26, 4.66)		
	Median (IQR)	25 (15, 35)	25 (10, 35)	25 (15, 35)			
	Min., Max.	0, 150	0, 115	0, 150			
**Overall**						3.02 (-0.03, 6.08)	0.052
**PHQ-9**							
**BL**	N (%)	285 (100%)	56 (100%)	341 (100%)			
	Mean (SD)	3.1 (3.5)	2.8 (3.2)	3.0 (3.4)	0.22 (-0.77, 1.21)		
	Median (IQR)	2 (1, 4)	2 (1, 4)	2 (1, 4)			
	Min., Max.	0, 23	0, 16	0, 23			
**FU1**	N (%)	270 (95%)	55 (98%)	325 (95%)			
	Mean (SD)	2.4 (2.8)	3.3 (4.3)	2.5 (3.1)	-0.92 (-1.81, -0.03)		
	Median (IQR)	2 (0, 3)	2 (0, 4)	2 (0, 3)			
	Min., Max.	0, 17	0, 17	0, 17			
**FU2**	N (%)	270 (95%)	55 (98%)	325 (95%)			
	Mean (SD)	2.4 (3.0)	2.8 (3.6)	2.5 (3.1)	-0.36 (-1.25, 0.53)		
	Median (IQR)	1 (1, 3)	1 (0, 4)	1 (0, 3)			
	Min., Max.	0, 20	0, 13	0, 20			
**FU3**	N (%)	269 (94%)	53 (95%)	322 (94%)			
	Mean (SD)	2.9 (3.5)	2.9 (3.7)	2.9 (3.5)	0.02 (-1.02, 1.06)		
	Median (IQR)	2 (1, 4)	2 (0, 4)	2 (0, 4)			
	Min., Max.	0, 21	0, 15	0, 21			
***Overall***						-0.61 (-1.34, 0.12)	0.100

Primary analysis found no significant differences between arms for the key secondary outcome of parent depression (Table 2) adjusted mean difference = -0.61; 95% CI (-1.34, 0.12); p = 0.1). Sensitivity increased the difference between groups (-0.64; CI (-1.35, 0.07); p = 0.077), but did not alter the primary analysis findings, see S6 Table.

Other secondary outcomes showed no arm differences on any measures including for how children were fed (e.g. breast, bottle, mixed. See S7 Table.

### Economic evaluation

E-SEE Steps had higher costs (£2,610 vs £1,989) and QALYs (2.618 vs 2.587) compared to SAU over the trial period, resulting in an ICER of £20,062 per QALY compared to services as usual (see Table 3).

**Table 3:** Cost-effectiveness results.

	Costs	Adult QALYs	Child QALYs	Overall QALYs	ICER	Probability of being cost-effective for given cost-effectiveness threshold		
	(95% CI)	(95% CI)	(95% CI)	(95% CI)		£15,000 per QALY	£20,000 per QALY	£30,000 per QALY
	[P(most costly)]	[P(most effective)]	[P(most effective)]	[P(most effective)]				
**Services as usual**	£1,988.61	1.31392	1.2742	2.5868		0.64	0.512	0.332
	(1465.79, 2615.43)	(1.275, 1.352)	(1.267, 1.282)	(2.549, 2.621)				
	[0.037]	[0.044]	[0.868]	[0.06]				
**E-SEE Steps**	£2,609.46	1.34818	1.26957	2.61775	£20,061 per QALY	0.36	0.488	0.668
	(2312.07, 2951.04)	(1.333, 1.364)	(1.266, 1.273)	(2.603, 2.634)				
	[0.963]	[0.956]	[0.132]	[0.94]				

The small gain in mean QALYs in adults outweighed minor decrements reported in child outcomes over the trial period. All scenarios found E-SEE Steps cost-effective at the maximum recommended threshold of £30,000 per QALY, see S8 Table. The probability of E-SEE Steps being cost-effective was estimated at 36%, 49% and 67% for £15,000, £20,000, and £30,000 cost-effectiveness thresholds, respectively.

Post-randomization adverse events (serious = 86; other = 96) adverse events (AEs) were recorded, and included injuries or conditions arising from childbirth, and common infant ailments such as bronchitis; all were unrelated to the intervention and there were no differences between arms regarding their proportion or nature.

## Discussion

The findings show no positive effect for E-SEE Steps on child social-emotional wellbeing at 20 months when compared to the control arm. ASQ:SE-2 scores declined (worsened) for both arms, but the intervention arm declined more. No significant effect was found for the key secondary outcome, parental depression; sensitivity analyses strengthened the signal in favor of the intervention, but it was not significant. No statistically significant effects were found for any secondary outcomes. Parent take-up of IY-T was lower than expected, and fidelity of delivery for IY-I and IY-T was mixed, both of which may have influenced the findings. The cost-effectiveness of E-SEE Steps was contingent on relatively modest differentials in parental health-related quality of life (HRQoL) that were short in duration, partially offset by reductions in child HRQoL.

This study is the first in the UK to explore the use of a proportionate, longitudinal, universal delivery model with a specific parent intervention. This trial showed no evidence of effectiveness for E-SEE Steps overall, and it was not possible to assess the IY-I or IY-T programs for effectiveness as ‘stand-alone’ interventions in this model. Other RCTs of stand-alone interventions to support child outcomes in the very early years have also failed to find an effect, e.g. [31] study of the Family Nurse Partnership trial, however this focused on parent outcomes during and after pregnancy (which is typically a pre-requisite for child outcome changes). Triple-P Baby is a program from a suite of Triple-P programs (https://www.triplep.net/glo-en/home/), as is Mellow Bumps (https://www.mellowparenting.org/). These programs are for parents during/after pregnancy and are currently undergoing trial. Although no effectiveness results have been published for these parent and baby programs the Mellow Bumps trial ‘THRIVE’ process evaluation findings suggest that vulnerable families did not benefit and felt marginalized, and that more is needed to support such families in attending the parent programs for the full duration [32]. However, a controlled trial in Ireland that investigated IY-I as part of a wraparound service (called the ‘Up2Two’) and found parenting efficacy and child cognitive stimulation effects [33]. Overall, more work is needed to identify effective parenting interventions for families with infants/toddlers.

This study had several key strengths. The proportionate universal trial design reflected real-world services addressed different familial levels of need. E-SEE Steps combined universal preventative and early intervention/treatment elements. Low levels of missing data and a high participant retention (94%) somewhat mitigated against not achieving target recruitment, retaining sufficient power to address the main research question. An independent observational outcome measure was used, in addition to parent report, and a robust measure selection strategy was undertaken.

However, the study was not powered to establish the effectiveness of *each* of the individual three E-SEE Steps (or four possible doses) with the sample. Low IY group numbers and attendance rates (and small control n) meant that planned secondary analysis to explore each level of intervention could not be conducted. Sample representativeness is also questionable; 45% of mothers had an undergraduate degree or higher (lower than the national average of 57%, see ONS data*)*, and 11% of parents were single/not in a “live-in” relationship (lower than the national average of 23–25%—according to 2019 Gingerbread and Office for National Statistics (see Table 1 in *Families and households*).

Despite careful measure selection, caution is needed in interpretation; the ASQ:SE-2 (which is routinely used in the UK for 24-month child developmental assessments) and the observational Infant Care Index are not validated in the UK. The SDQ (2-4-yr Version) [19] is the is not validated for the trial age-group (20-months-old), but we used the youngest age SDQ version available; Infant Care Index analysis was conducted on a subset with complete data at all timepoints. The lack of appropriate and robust measures across infancy and toddlerhood [13] highlights an important need for more psychometric studies in this area.

The confidence intervals in the sensitivity analysis included an MCID of +5 on the ASQ:SE-2, which could be considered as the opposite of positive clinical effect which we defined as -5, although we have insufficient information on which to base this claim. Long-term outcomes could not be measured within the trial period.

The low conversion rates from eligibility to accessing at least one session (IY-I = 50%, IY-T = 21%) could suggest difficulties in engaging parents, or that the program was not attractive to parents, and/or they were too overwhelmed to participate. We found that parents with higher levels of depressive symptoms were less likely to attend IY-I. Mental health provision during pregnancy and the perinatal period in the UK remains limited, despite NICE guidance [34] and the potential negative impact on children. It is possible that more engagement work with families is needed to encourage take-up, or to offer families alternative supports as appropriate. The lower take-up of IY-T could also reflect a return to work and greater flexibility, therefore, on the timing of group delivery, may be needed.

Less than half of parents who attended the targeted programs completed them, although 80% were still attending at week 5, suggesting that parents may prefer/can commit to shorter programs. Low uptake and retention rates likely impacted the findings and this, combined with varying levels of fidelity, suggests that system and possibly program changes may be needed (Berry et al., submitted). A pre-intervention component to identify, engage and retain parents, and those with low mood, may help to reduce attendance barriers [35]. Given the uncertainty around long-term parental and child outcomes, the cost-effectiveness of E-SEE Steps remains equivocal.

Although IY-I and IY-T could not be individually assessed for effectiveness in this study, IY-B will be explored by combining pilot [11] and main trial data. We expected similar (not different) trajectories for the primary and key secondary outcomes given the relationship between parent mental health and child social emotional wellbeing. A longer-term follow-up could explore whether E-SEE works preventatively or not, i.e. intervention family outcomes are sustained but control families “worsen” in comparison.

## Conclusions

E-SEE Steps, a proportionate universal (stepped) delivery model of a program for parents of infants and toddlers was challenging to implement, had lower than expected parental uptake for IY-T, and was not effective in enhancing child social emotional wellbeing or reducing parent depression.

E-SEE Steps was borderline cost-effective over the period of the trial, but cost-effectiveness over the longer term will depend on the persistence of modest effects on parent mental health.

Collectively, the findings suggest that the current model cannot yet be recommended for use. Changes to the delivery systems, and to the individual programs within the model, may be needed prior to any future trials of this model.

The evidence gap for parent programs for children under age two to enhance child social emotional wellbeing remains, and further research is needed to establish the most appropriate means to support early child wellbeing in a preventive and indicated way.

## Supporting information

S1 Fig E-SEE steps model. (DOCX)

S2 Fig Independent vs self-report adherence. (DOCX)

S1 Table CONSORT checklist. (DOCX)

S2 Table Summary of incredible years content. (DOCX)

S3 Table Eligibility and take-up. (DOCX)

S4 Table Comparing attendees and non-attendees. (DOCX)

S5 Table Attendance by ASQ:SE-2 and PHQ-9. (DOCX)

S6 Table Summary of sensitivity analysis. (DOCX)

S7 Table Arm differences for secondary outcomes. (DOCX)

S8 Table Unit costs_cost-effectiveness analysis. (DOCX)

S1 Text Economic sensitivity analysis. (DOCX)

## References

[1] Burt SA, Hyde LW, Frick PJ, Jaffee SR, Shaw DS, Tremblay R. Commentary: Childhood conduct problems are a public health crisis and require resources: a commentary on Rivenbark. JCCP. 2018;59: 711–713. doi: 10.1111/jcpp.12930 29808490

[2] Hurt L, Paranjothy S, Lucas PJ, Watson D, Mann M, Griffiths LJ, et al. Interventions that enhance health services for parents and infants to improve child development and social and emotional well-being in high-income countries: a systematic review. BMJ Open. 2018;8, e014899. doi: 10.1136/bmjopen-2016-014899 29439064 PMC5829600

[3] Furlong M, McGilloway S, Bywater T, Hutchings J, Smith SM, Donnelly M. Behavioural and cognitive-behavioural group-based parenting programmes for early-onset conduct problems in children aged 3 to 12 years. Cochrane Database Syst Rev. 2021; 2, CD008225–CD. 10.1002/14651858.CD008225.pub2 PMC12935172 22336837

[4] Pidano AE, Allen AR. The Incredible Years series: A review of the independent research base. J Child Fam Stud. 2015;24: 1898–1916.

[5] Leijten P, Gardner F, Melendez-Torres GJ, Weeland J, Hutchings J, Landau S, et al. Co-occurring change in children’s conduct problems and maternal depression: Latent class individual participant data meta-analysis of the Incredible Years parenting program. Dev Psychopathol. 2019;31: 1851–1862. doi: 10.1017/S0954579419001068 31370916

[6] Pietikäinen JT, Kiviruusu O, Kylliäinen A, Pölkki P, Saarenpää-Heikkilä O, Paunio T, et al. Maternal and paternal depressive symptoms and children’s emotional problems at the age of 2 and 5 years: a longitudinal study. JCPP. 2020;61: 195–204. doi: 10.1111/jcpp.13126 31535379

[7] Jones CH, Erjavec M, Viktor S, Hutchings J. Outcomes of a comparison study into a group-based infant parenting programme. J Child Fam Stud. 2016;25: 3309–3321. doi: 10.1007/s10826-016-0489-3 27795658 PMC5061836

[8] Hutchings J, Griffith N, Bywater T, Williams ME, Baker-Henningham H. Targeted vs universal provision of support in high-risk communities: comparison of characteristics in two populations recruited to parenting interventions. J Child Serv. 2013;8: 169–182.

[9] Marmot M, Allen J, Goldblatt P, Boyce T, McNeish D, Grady M, et al. The Marmot review: Fair society, healthy lives. Strategic review of health inequalities in England post-2010 London; 2010. The Marmot Review. December 11, 2020 https://www.instituteofhealthequity.org/resources-reports/fair-society-healthy-lives-the-marmot-review

[10] Candlish J, Teare MD, Cohen J, Bywater T. Statistical design and analysis in trials of proportionate interventions: a systematic review. Trials. 2019:20,151. doi: 10.1186/s13063-019-3206-x 30819224 PMC6396459

[11] Blower S, Berry L, Bursnall M, Cohen J, Gridley N, Loban A, et al. Enhancing Social-Emotional Outcomes in Early Years (E-SEE): Randomized Pilot Study of Incredible Years Infant and Toddler Programs. J Child Fam Stud. 2021;30: 1933–1949 (2021). doi: 10.1007/s10826-021-01991-7

[12] Bywater TJ, Berry V, Blower SL, Cohen J, Gridley N, Kiernan K, et al. Enhancing social-emotional health and wellbeing in the early years (E-SEE). A study protocol of a community-based randomised controlled trial with process and economic evaluations of the Incredible Years infant and toddler parenting programmes, delivered in a Proportionate Universal Model. BMJ Open. 2018:8, e026906. doi: 10.1136/bmjopen-2018-026906 30573493 PMC6303737

[13] Gridley N, Blower SL, Dunn AC, Bywater TJ, Whittaker K, Bryant MJ. Psychometric Properties of Child (0–5 Years) Outcome Measures as used in Randomized Controlled Trials of Parent Programs: a Systematic Review. Clin Child Fam Psychol Rev. 2019;22: 388–405. doi: 10.1007/s10567-019-00277-1 30806864 PMC6669186

[14] Blower SL, Gridley N, Dunn A, Bywater T, Hindson Z, Bryant M. Psychometric Properties of parent outcome measures used in RCTs of antenatal and early years parent programs: a systematic review. Clin Child Fam Psychol Rev. 2019;22: 367–387. doi: 10.1007/s10567-019-00276-2 30796674 PMC6669247

[15] Squires J, Bricker D, Twombly E. ASQ:SE-2 User’s guide (2nd ed.) Baltimore: Paul Brookes Publishing Company; 2015.

[16] Kroenke K, Spitzer RL, Williams JBW. The PHQ-9: validity of a brief depression severity measure. J Gen Intern Med. 2001;16: 606–613. doi: 10.1046/j.1525-1497.2001.016009606.x 11556941 PMC1495268

[17] Johnston C, Mash EJ. A measure of parenting satisfaction and efficacy. J Clin Child Psychol. 1989; 18: 167–175.

[18] Crittenden, P.M. (2010). CARE-Index infancy: coding manual. Miami, FL, USA.

[19] Goodman R. The strengths and difficulties questionnaire: A research note. JCCP. 1997;38: 581–586. doi: 10.1111/j.1469-7610.1997.tb01545.x 9255702

[20] Condon JT, Corkindale CJ. The assessment of parent-to-infant attachment: Development of a self-report questionnaire instrument. J Reprod Infant Psychol. 1998;16: 57–76.

[21] Bandura A, Walters RH. Social learning theory. Prentice-hall Englewood Cliffs, NJ; 1977.

[22] Bowlby J. A Secure base: parent-child attachment and healthy human development. New York: Basic Books, Inc; 1988.

[23] Bywater T, Gridley N, Berry V, Blower S, Tobin K. The parent programme implementation checklist (PPIC): The development and testing of an objective measure of skills and fidelity for the delivery of parent programmes. CCiP. 2019;25: 281–309.

[24] Berry V, Mitchell SB, Blower SL, Whittaker K, Wilkinson K, McGilloway S, et al. Barriers and Facilitators in Proportionate Universal Parenting Support in Community Family Services: A Process Evaluation of the Incredible Years^®^ Infant and Toddler Parenting Programs (E-SEE Steps). PLos ONE. Forthcoming 2021. 10.1371/journal.pone.0265946 PMC9191704 35696375

[25] Beecham J, Knapp MRJYN. Costing psychiatric interventions. 2nd edn. In Thornicroft G., Wing J., & Brewin C. R. (Eds.), Measuring mental health needs (pp. 200–224). London: Gaskell/Royal College of Psychiatrists; 1992.

[26] Varni JW, Seid M, Rode CA. The PedsQL: Measurement model for the pediatric quality of life inventory. Med Care. 1999;37: 126–139. doi: 10.1097/00005650-199902000-00003 10024117

[27] Essink-Bot ML, Krabbe PF, Bonsel GJ, Aaronson NK. An empirical comparison of four generic health status measures. The Nottingham Health Profile, the Medical Outcomes Study 36-item Short-Form Health Survey, the COOP/WONCA charts, and the EuroQol instrument. Med Care. 1997;35: 522–537. doi: 10.1097/00005650-199705000-00008 9140339

[28] Furber G, Segal L, Leach M, Cocks J. Mapping scores from the Strengths and Difficulties Questionnaire (SDQ) to preference-based utility values. Qual Life Res. 2014;23: 403–411. doi: 10.1007/s11136-013-0494-6 23943259

[29] Buuren S van Groothuis-Oudshoorn K. ‘MICE: Multivariate Imputation by Chained Equations in R’. J Stat Softw. 2011;45(3): 1–67.

[30] National Institute of Health and Care Excellence (NICE). Guide to the methods of technology appraisal [PMG9]. 2013. December 11, 2020 https://www.nice.org.uk/process/pmg9/chapter/foreword 27905712

[31] Robling M, Bekkers MJ, Bell K, Butler CC, Cannings-John R, Channon S, et al. Effectiveness of a nurse-led intensive home-visitation programme for first-time teenage mothers (Building Blocks): a pragmatic randomised controlled trial. Lancet. 2016;387: 146–155. doi: 10.1016/S0140-6736(15)00392-X 26474809 PMC4707160

[32] Buston K, O’Brien R, Wight D, Henderson M. The Lancet The reflective component of the Mellow Bumps parenting intervention: Implementation, engagement and mechanisms of change. PLoS ONE. 2019. 14(4): e0215461. doi: 10.1371/journal.pone.0215461 30990855 PMC6467403

[33] Hickey G, McGilloway S, Leckey Y, Leavy S, Stokes A., O’Connor S. et al. Exploring the potential utility and impact of a universal, multi-component early parenting intervention through a community-based, controlled trial. Child Youth Serv Rev. 2020;118: 105458.

[34] National Institute for Health and Care Excellence (NICE). Children’s attachment: attachment in children and young people who are adopted from care, in care or at high risk of going into care. NICE guideline [NG26]. 2015. December 11, 2020 https://www.nice.org.uk/guidance/ng26 26741018

[35] Nock MK, Kazdin AE. Randomized controlled trial of a brief intervention for increasing participation in parent management training. J Consult Clin Psychol. 2005;73(5): 872–879. doi: 10.1037/0022-006X.73.5.872 16287387

