# Supplementary material for: A randomized controlled trial of a proportionate universal parenting program delivery model (E-SEE Steps) to enhance child social-emotional wellbeing
Source: PLoS One. 2022 Apr 4;17(4):e0265200. doi: 10.1371/journal.pone.0265200 (PMC8979462; doi:10.1371/journal.pone.0265200)
Supplement: S1 Fig — (DOCX) [file pone.0265200.s001.docx]

**S1 Fig. E-SEE Steps Model**

**Targeted level 9 months post-baseline**: children are approx. 11-12 months. **SOME** parents are offered the 12-week Incredible Years Toddler (IY-T) group program.

Targeted level offer depends on parent rating of their, or their childs, wellbeing

**Targeted level 2 months post-baseline**: Infants are approx. 4 months. **SOME** parents are offered the 10-week Incredible Years Infant (IY-I) group program.

**Universal level:** **ALL** parents randomised to intervention receive the Incredible Years Baby Book (IY-B) post baseline and randomisation. Infants are approx. 2 months old.
