# Supplementary material for: A randomized controlled trial of a proportionate universal parenting program delivery model (E-SEE Steps) to enhance child social-emotional wellbeing
Source: PLoS One. 2022 Apr 4;17(4):e0265200. doi: 10.1371/journal.pone.0265200 (PMC8979462; doi:10.1371/journal.pone.0265200)
Supplement: S2 Fig — (DOCX) [file pone.0265200.s002.docx]

**S2 Fig. Independent Vs self-report adherence.**

*Note. There was only one IY-T group for Site 3 and Site 4 (labelled accordingly). Site 3 and 4 had two IY-I groups split as 3a and 3b, and 4a and 4b respectively.*
