# Supplementary material for: A randomized controlled trial of a proportionate universal parenting program delivery model (E-SEE Steps) to enhance child social-emotional wellbeing
Source: PLoS One. 2022 Apr 4;17(4):e0265200. doi: 10.1371/journal.pone.0265200 (PMC8979462; doi:10.1371/journal.pone.0265200)
Supplement: S3 Table — (DOCX) [file pone.0265200.s005.docx]

**S3 Table. Eligibility and take-up**

|  | **Program** | **Number Eligible (% of intervention arm)** | **Number accepted (% of those eligible)** | **Number attending at least one session** |
| --- | --- | --- | --- | --- |
|  |  |  |  |  |
| **Site 1** | IY-I | 35/105 (33%) | 20 (57%) | 14 |
| **Site 2** | IY-I | 24/71 (34%) | 21 (88%) | 15 |
| **Site 3** | IY-I | 17/50 (34%) | 13 (77%) | 13 |
| **Site 4** | IY-I | 25/59 (42%) | 11 (44%) | 9 |
| **Site 1** | IY-T | 32/105 (30%) | 12 (38%) | 4 |
| **Site 2** | IY-T | 26/71 (37%) | 15 (58%) | 7 |
| **Site 3** | IY-T | 17/50 (34%) | 5 (29%) | 2 |
| **Site 4** | IY-T | 26/59 (44%) | 16 (62%) | 8 |
| **Total** |  | **202** | **113 (56%)** | **72** |
