# Supplementary material for: A randomized controlled trial of a proportionate universal parenting program delivery model (E-SEE Steps) to enhance child social-emotional wellbeing
Source: PLoS One. 2022 Apr 4;17(4):e0265200. doi: 10.1371/journal.pone.0265200 (PMC8979462; doi:10.1371/journal.pone.0265200)
Supplement: S4 Table — (DOCX) [file pone.0265200.s006.docx]

**S4A Table. Comparing attendees and non-attendees**

| **IY-I** |  |  |  |
| --- | --- | --- | --- |
|  |  | **Did not attend** | **Attended** |
| **ASQ:SE-2 (development) FU1** | Below monitoring zone (n=22) | 12 (26%) | 10 (20%) |
|  | In monitoring zone (n=17) | 7 (15%) | 10 (20%) |
|  | In refer zone (n=58) | 28 (60%) | 30 (60%) |
|  |  |  |  |
| **PHQ-9 (Depression) FU1** | None (n=53) | 23 (46%) | 30 (59%) |
|  | Mild (n=37) | 22 (44%) | 15 (29%) |
|  | Moderate and moderately severe (n=11) | 5 (10%) | 6 (12%) |
|  |  |  |  |
| **Whether qualified to degree level** | Below Degree Level (n=37) | 22 (44%) | 15 (29%) |
|  | Degree level or Higher (n=64) | 28 (56%) | 36 (71%) |
|  |  |  |  |
| **Quartile of IY-I eligible participants weekly income** | lowest quartile (<360) (n=20) | 11 (22%) | 9 (18%) |
|  | 2nd quartile (between 360 and 600) (n=25) | 14 (28%) | 11 (22%) |
|  | 3rd quartile (between 600 and 900) (n=16) | 8 (16%) | 8 (16%) |
|  | Highest quartile (> 900) (n=19) | 6 (12%) | 13 (25%) |
|  | Income not given (n=21) | 11 (22%) | 10 (20%) |
|  |  |  |  |
| **Whether first child** | Not first child (n=56) | 27 (54%) | 29 (57%) |
|  | First child (n=45) | 23 (46%) | 22 (43%) |
|  |  |  |  |
| **Sex of Child** | Male (n=55) | 30 (60%) | 25 (49%) |
|  | Female (n=46) | 20 (40%) | 26 (51%) |
|  |  |  |  |
| **Site** | Site 3 (n=17) | 4 (8%) | 13 (25%) |
|  | Site 2 (n=24) | 9 (18%) | 15 (29%) |
|  | Site 4 (n=25) | 16 (32%) | 9 (18%) |
|  | Site 1 (n=35) | 21 (42%) | 14 (27%) |
|  |  |  |  |
| **Relationship status** | Married and living together (n=65) | 31 (62%) | 34 (67%) |
|  | Cohabiting / living together (n=20) | 11 (22%) | 9 (18%) |
|  | Other type of relationship (n=8) | 4 (8%) | 4 (8%) |
|  | Not in a relationship or separated (n=8) | 4 (8%) | 4 (8%) |
|  |  |  |  |
| **Parent's age group** | 18 to 21 (n=6) | 4 (8%) | 2 (4%) |
|  | 22 to 25 (n=17) | 10 (20%) | 7 (14%) |
|  | 26 to 30 (n=23) | 11 (22%) | 12 (24%) |
|  | 31 to 35 (n=35) | 20 (40%) | 15 (29%) |
|  | 36 and above (n=20) | 5 (10%) | 15 (29%) |
|  |  |  |  |
| **Ethnicity** | English/Welsh/Scottish/Northern Irish/British/Irish (n= 73) | 32 (64%) | 41 (80%) |
|  | Any other White background (n=4) | 3 (6%) | 1 (2%) |
|  | Indian (n=9) | 7 (14%) | 2 (4%) |
|  | Pakistani (n=6) | 3 (6%) | 3 (6%) |
|  | Any Other ethnic group (n=9) | 5 (10%) | 4 (8%) |

**S4B Table. Comparing attendees and non-attendees**

| **IY-T** |  |  |  |
| --- | --- | --- | --- |
|  |  | Did not attend | Attended |
| **ASQ:SE-2 (development) FU1** | Below monitoring zone (n=42) | 32 (48%) | 10 (53%) |
|  | In monitoring zone (n=12) | 10 (15%) | 2 (11%) |
|  | In refer zone (n=32) | 25 (37%) | 7 (37%) |
|  |  |  |  |
| **PHQ-9 (Depression) FU1** | None (n=62) | 52 (67%) | 10 (53%) |
|  | Mild (n=27) | 21 (27%) | 6 (32%) |
|  | Moderate and moderately severe (n=8) | 5 (6%) | 3 (16%) |
|  |  |  |  |
| **Whether qualified to degree level** | Below Degree Level (n=38) | 28 (35%) | 10 (48%) |
|  | Degree level or Higher (n=63) | 52 (65%) | 11 (52%) |
|  |  |  |  |
| **Quartile of IY-T eligible participants weekly income di** | lowest quartile (<450) (n=20) | 14 (18%) | 6 (29%) |
|  | 2nd quartile (between 450 and 610) (n=20) | 16 (20%) | 4 (19%) |
|  | 3rd quartile (between 610 and 1000) (n=21) | 19 (24%) | 2 (10%) |
|  | Highest quartile (> 1000) (n=18) | 14 (18%) | 4 (19%) |
|  | Income not given (n=22) | 17 (21%) | 5 (24%) |
|  |  |  |  |
| **Whether first child** | Not first child (n=57) | 44 (55%) | 13 (62%) |
|  | First child (n=44) | 36 (45%) | 8 (38%) |
|  |  |  |  |
| **Sex of Child** | Male (n=59) | 45 (56%) | 14 (67%) |
|  | Female (n=42) | 35 (44%) | 7 (33%) |
|  |  |  |  |
| **Site** | Site 3 (n=17) | 15 (19%) | 2 (10%) |
|  | Site 2 (n=26) | 19 (24%) | 7 (33%) |
|  | Site 4 (n=26) | 18 (23%) | 8 (38%) |
|  | Site 1 (n=32) | 28 (35%) | 4 (19%) |
|  |  |  |  |
| **Relationship status** | Married and living together (n=65) | 53 (66%) | 12 (57%) |
|  | Cohabiting / living together (n=22) | 17 (21%) | 5 (24%) |
|  | Other type of relationship (n=7) | 6 (8%) | 1 (5%) |
|  | Not in a relationship or separated (n=7) | 4 (5%) | 3 (14%) |
|  |  |  |  |
| **Parent's age group** | 18 to 21 (n=4) | 2 (3%) | 2 (10%) |
|  | 22 to 25 (n=15) | 11 (14%) | 4 (19%) |
|  | 26 to 30 (n=26) | 23 (29%) | 3 (14%) |
|  | 31 to 35 (n=32) | 26 (33%) | 6 (29%) |
|  | 36 and above (n=24) | 18 (23%) | 6 (29%) |
|  |  |  |  |
| **Ethnicity** | English/Welsh/Scottish/Northern Irish/British/Irish (n= | 64 (80%) | 17 (81%) |
|  | Any other White background (n=0) | 0 (0%) | 0 (0%) |
|  | Indian (n=7) | 6 (8%) | 1 (5%) |
|  | Pakistani (n=6) | 5 (6%) | 1 (5%) |
|  | Any Other ethnic group (n=7) | 5 (6%) | 2 (10%) |
