# Supplementary material for: A randomized controlled trial of a proportionate universal parenting program delivery model (E-SEE Steps) to enhance child social-emotional wellbeing
Source: PLoS One. 2022 Apr 4;17(4):e0265200. doi: 10.1371/journal.pone.0265200 (PMC8979462; doi:10.1371/journal.pone.0265200)
Supplement: S5 Table — (DOCX) [file pone.0265200.s007.docx]

**S5 Table. Attendance by ASQ:SE-2 and PHQ-9**

|  | **Treatment** |  |  | **Control** |  |  |  |  |  |
| --- | --- | --- | --- | --- | --- | --- | --- | --- | --- |
|  | n (%) | Mean | SD | n (%) | Mean | SD | Mean diff (95% CI) | Adjusted Mean diff (95% CI) | p-value |
| **Baseline** |  |  |  |  |  |  |  |  |  |
| **FU0** | 101 (100%) | 32.62 | 17.60 | 19 (100%) | 33.16 | 22.93 | -0.53 (-9.60, 8.54) |  |  |
| **Outcome** |  |  |  |  |  |  |  |  |  |
| **FU2** | 97 (100%) | 36.86 | 17.17 | 19 (100%) | 35.00 | 12.58 | 1.86 (-6.27, 9.99) |  |  |
| **Overall** |  |  |  |  |  |  |  | 1.90 (-5.93, 9.73) | 0.63 |
| **Baseline** |  |  |  |  |  |  |  |  |  |
| **FU0** | 101 (100%) | 28.51 | 17.51 | 22 (100%) | 27.95 | 18.62 | 0.56 (-7.61, 8.73) |  |  |
| **FU3** | 99 (100%) | 35.40 | 22.23 | 21 (100%) | 37.86 | 24.63 | -2.45 (-13.12, 8.22) |  |  |
| **Overall** |  |  |  |  |  |  |  | -0.85 (-12.09, 10.39) | 0.88 |
