# Supplementary material for: A randomized controlled trial of a proportionate universal parenting program delivery model (E-SEE Steps) to enhance child social-emotional wellbeing
Source: PLoS One. 2022 Apr 4;17(4):e0265200. doi: 10.1371/journal.pone.0265200 (PMC8979462; doi:10.1371/journal.pone.0265200)
Supplement: S6 Table — (DOCX) [file pone.0265200.s008.docx]

**S6 Table.** **Summary of sensitivity analysis**

|  | **Adjusted Mean diff (95% CI)** | **p-value** |
| --- | --- | --- |
| **ASQ:SE-2 Primary Analysis model** | 3.02 (-0.03, 6.08) | 0.052 |
| **ASQ:SE-2 Sensitivity Analyses** |  |  |
| Z transformation | 0.19 (0.01, 0.36) | 0.036* |
| Percentage transformation | 0.81 (0.06, 1.56) | 0.035* |
| Primary with unstructured correlation | 2.56 (-0.69, 5.80) | 0.122 |
| Permutation test |  | 0.069 |
| Difference from baseline - multiple imputation | 1.97 (-1.22, 5.17) | 0.23 |
| **PHQ-9 Primary Analysis model** | -0.61 (-1.34, 0.12) | 0.100 |
| **PHQ-9 Sensitivity Analyses** |  |  |
| Primary with unstructured correlation | -0.64 (-1.35, 0.07) | 0.077 |
| Difference from baseline - multiple imputation | -0.68 (-1.37, 0.12) | 0.054 |

*significant at the 0.05 level
