# Supplementary material for: A randomized controlled trial of a proportionate universal parenting program delivery model (E-SEE Steps) to enhance child social-emotional wellbeing
Source: PLoS One. 2022 Apr 4;17(4):e0265200. doi: 10.1371/journal.pone.0265200 (PMC8979462; doi:10.1371/journal.pone.0265200)
Supplement: S7 Table — (DOCX) [file pone.0265200.s009.docx]

**S7A Table.** **Difference between arms for secondary outcomes**

|  | **Treatment** |  |  | **Control** |  |  | **Differences** |  |
| --- | --- | --- | --- | --- | --- | --- | --- | --- |
|  | **n (%)** | **Mean** | **SD** | **n (%)** | **Mean** | **SD** | **Mean diff (95% CI)** | **Adjusted Mean diff (95% CI) p-value** |
| **Infant CARE-Index** |  |  |  |  |  |  |  |  |
| **BL** | 105 (100%) | 5.87 | 2.48 | 14 (100%) | 5.57 | 2.14 | 0.30 (-1.06, 1.66) |  |
| **FU1** | 105 (100%) | 6.10 | 2.62 | 14 (100%) | 7.79 | 3.07 | -1.69 (-3.18, -0.20) |  |
| **FU2** | 105 (100%) | 8.18 | 2.79 | 14 (100%) | 8.07 | 2.70 | 0.11 (-1.44, 1.66) |  |
| **FU3** | 105 (100%) | 8.33 | 2.59 | 14 (100%) | 7.93 | 2.50 | 0.40 (-1.04, 1.84) |  |
| **Overall** |  |  |  |  |  |  |  | -0.25 (-1.09,0.59) 0.56 |
| **PSOC** |  |  |  |  |  |  |  |  |
| **BL** | 285 (100%) | 80.94 | 9.56 | 56 (100%) | 80.21 | 9.76 | 0.72 (-2.03, 3.47) |  |
| **FU1** | 270 (95%) | 82.81 | 9.66 | 55 (98%) | 81.84 | 10.63 | 0.97 (-1.88, 3.82) |  |
| **FU2** | 270 (95%) | 83.84 | 8.90 | 55 (98%) | 82.69 | 10.00 | 1.15 (-1.49, 3.79) |  |
| **FU3** | 269 (94%) | 81.88 | 9.41 | 53 (95%) | 82.43 | 9.70 | -0.56 (-3.35, 2.23) |  |
| **Overall** |  |  |  |  |  |  |  | 0.07 (-1.74, 1.87) 0.94 |
| **EQ5D** |  |  |  |  |  |  |  |  |
| **BL** | 285 (100%) | 0.94 | 0.08 | 56 (100%) | 0.92 | 0.10 | 0.02 (-0.00, 0.04) |  |
| **FU1** | 270 (95%) | 0.95 | 0.08 | 55 (98%) | 0.93 | 0.10 | 0.03 (0.01, 0.05) |  |
| **FU2** | 270 (95%) | 0.95 | 0.09 | 55 (98%) | 0.92 | 0.12 | 0.03 (0.00, 0.06) |  |
| **FU3** | 269 (94%) | 0.94 | 0.10 | 53 (95%) | 0.93 | 0.10 | 0.01 (-0.02, 0.04) |  |
| **Overall** |  |  |  |  |  |  |  | 0.02 (0.00, 0.04) 0.04 |
| **Difference between arms for outcomes at 18 months (FU3) only** | | | | | | | | |
| **SDQ** | 266 (99%) | 9.67 | 4.27 | 53 (100%) | 9.15 | 4.53 | 0.52 (-0.75, 1.79) | 0.64 (-0.64, 1.91) 0.33 |
| **PEDSQL** | 268 (100%) | 86.70 | 9.61 | 53 (100%) | 87.31 | 9.15 | -0.61 (-3.42, 2.20) | -0.60 (-3.22, 2.01) 0.65 |
| **MPAS** | 257 (96%) | 84.61 | 5.82 | 52 (98%) | 84.27 | 7.15 | 0.34 (-1.47, 2.15) | 0.94 (-0.76, 2.64) 0.28 |

**S7B Table.** **Difference Between Arms for Secondary Outcomes**

| **Infant feeding up to 12 months** |  | **Treatment** | **Control** | **All** |
| --- | --- | --- | --- | --- |
|  |  | **n=285** | **N=51** | **N=341** |
| **BL (< 10 weeks old)** |  |  |  |  |
| Currently breastfeeding |  | 179 (63%) | 32 (57%) | 211 (62%) |
| Never breastfed |  | 31 (11%) | 11 (20%) | 42 (12%) |
| previously breastfed |  | 75 (26%) | 13 (23%) | 88 (26%) |
| Of those currently breastfeeding |  |  |  |  |
|  | Also uses bottle | 88 (49%) | 14 (44%) | 102 (48%) |
|  | Does not use bottle | 91 (51%) | 18 (56%) | 109 (52%) |
| **FU1 (approx. 6 mos old)** |  |  |  |  |
| Currently breastfeeding |  | 149 (52%) | 28 (50%) | 177 (52%) |
| Never breastfed |  | 29 (10%) | 10 (18%) | 39 (11%) |
| Previously breastfed |  | 88 (31%) | 17 (30%) | 105 (31%) |
| Childs age in weeks when  Stopped breastfeeding | N (%) | 86 (98%) | 16 (94%) | 102 (97%) |
|  | Mean (SD) | 3.9 (4) | 5.6 (4) | 4.2 (4) |
|  | Median (IQR) | 3 (1, 6) | 5 (3, 8) | 3 (1, 6) |
|  | Min., Max. | 0, 13 | 0, 13 | 0, 13 |
| **FU2 (approx.12 mos old)** |  |  |  |  |
| Breast milk by breast |  | 110 (39%) | 19 (34%) | 129 (38%) |
| Breast milk by Bottle |  | 10 (4%) | 3 (5%) | 13 (4%) |
| Formula milk by bottle |  | 175 (61%) | 36 (64%) | 211 (62%) |
