# Supplementary material for: A randomized controlled trial of a proportionate universal parenting program delivery model (E-SEE Steps) to enhance child social-emotional wellbeing
Source: PLoS One. 2022 Apr 4;17(4):e0265200. doi: 10.1371/journal.pone.0265200 (PMC8979462; doi:10.1371/journal.pone.0265200)
Supplement: S8 Table — (DOCX) [file pone.0265200.s010.docx]

**S8A Table. Unit costs**

| **Resource** | **Unit cost** | | | **Source** | | | |
| --- | --- | --- | --- | --- | --- | --- | --- |
|  | **Average** | **Min^*^** | **Max^*^** |  |  |  |  |
| **E-SEE Steps Programme** |  |  |  |  | | | |
| ***IY Infant*** |  |  |  |  | | | |
| **Between-programme fixed costs** |  |  |  |  | | | |
| IY trainer fees & expenses | £2,842.50 | £1,555.71 | £4,211.18 | Recorded trial cost | | | |
| Venue hire with catering | £602.35 | £172.80 | £1,330.01 | Recorded trial cost | | | |
| Other equipment/materials | £100.00 | - | - | Recorded trial cost | | | |
| IY book (for group leaders) | £20.00 | - | - | Recorded trial cost | | | |
| Workshop manuals | £20.00 | - | - | Recorded trial cost | | | |
| Programme trainee fees | £16.20 | - | - | Recorded trial cost | | | |
| Leader manual and DVD set | £600.00 | - | - | Recorded trial cost | | | |
| Telephone supervision of team leaders | £763.81 | £330.00 | £1,294.24 | Recorded trial cost | | | |
| **Within-programme fixed costs** |  |  |  |  | | | |
| IY staff delivery costs (per hour) |  |  |  |  | | | |
| Mental health practitioners | £45.00 | - | - | Personal Social Services Research Unit, 2019 | | | |
| Early years worker / parenting coordinator | £62.53 | - | - | Personal Social Services Research Unit, 2018 | | | |
| Family support worker | £56.84 | - | - | Personal Social Services Research Unit, 2017 | | | |
| Child health worker | £61.50 | - | - | Personal Social Services Research Unit, 2018 | | | |
| Health visitor | £72.56 | - | - | Personal Social Services Research Unit, 2015 | | | |
| Community health nurse | £71.46 | - | - | Personal Social Services Research Unit, 2015 | | | |
| Clinical team lead | £87.00 | - | - | Personal Social Services Research Unit, 2019 | | | |
| Equipment/Materials (for up to 12 participants) | £200.00 | - | - | Recorded trial cost | | | |
| Catering (each session for up to 12 participants) | £57.32 | - | - | Recorded trial cost | | | |
| **Programme variable costs** |  |  |  |  | | | |
| IY book | £20.00 | - | - | Recorded trial cost | | | |
| Child centre/creche venue costs (per hour) | £15 | - | - | Recorded trial cost | | | |
| Other forms of contact |  |  |  |  | | | |
| Contact by phone | £6.00 | - | - | Personal Social Services Research Unit, 2019 | | | |
| Contact by home visit | £42.88 | - | - | Personal Social Services Research Unit, 2010 | | | |
| Contact by letter | £2.39 | - | - | Gidlow et al (2019) (22) | | | |
| Contact by text | £0.00 | - | - | Assumed cost-free as texts are already being sent | | | |
| ***IY Toddler*** |  |  |  |  | | | |
| **Between-programme fixed costs** |  |  |  |  | | | |
| Practitioner training costs |  |  |  |  | | | |
| IY trainer fees and expenses | £3,014.88 | £2,146.00 | £4,797.50 | Recorded trial cost | | | |
| Venue hire with catering | £491.19 | £250.00 | £588.00 | Recorded trial cost | | | |
| Other equipment/materials | £100.00 | - | - | Recorded trial cost | | | |
| IY book | £16.20 | - | - | Recorded trial cost | | | |
| Workshop manuals | £20.00 | - | - | Recorded trial cost | | | |
| Programme trainee fees | £16.20 | - | - | Recorded trial cost | | | |
| Leader manual and DVD set | £750.00 | - | - | Recorded trial cost | | | |
| Telephone supervision of team leaders | £1,312.38 | £552.50 | £2,687.00 | Recorded trial cost | | | |
| **Within-programme fixed costs** |  |  |  |  | | | |
| IY staff delivery costs (per hour) |  |  |  |  | | | |
| Mental health practitioners | £45.00 | - | - | Personal Social Services Research Unit, 2019 | | | |
| Early years worker / parenting coordinator | £62.53 | - | - | Personal Social Services Research Unit, 2018 | | | |
| Family support worker | £56.84 | - | - | Personal Social Services Research Unit, 2017 | | | |
| Child health worker | £61.50 | - | - | Personal Social Services Research Unit, 2018 | | | |
| Health visitor | £72.56 | - | - | Personal Social Services Research Unit, 2015 | | | |
| Community health nurse | £71.46 | - | - | Personal Social Services Research Unit, 2015 | | | |
| Clinical team lead | £87.00 | - | - | Personal Social Services Research Unit, 2019 | | | |
| Equipment/Materials (for up to 12 participants) | £200.00 | - | - | Recorded trial cost | | | |
| Catering (each session for up to 12 participants) | £57.32 | - | - | Recorded trial cost | | | |
| **Programme variable costs** |  |  |  |  | | | |
| IY book | £20.00 | - | - | Recorded trial cost | | | |
| Child centre/creche venue costs (per hour) | £15.00 | - | - | Recorded trial cost | | | |
| Contact by phone | £6.00 | - | - | Personal Social Services Research Unit, 2019 | | | |
| Contact by home visit | £42.88 | - | - | Personal Social Services Research Unit, 2010 | | | |
| Contact by letter | £2.39 | - | - | Gidlow et al (2019) | | | |
| Contact by text | £0.00 | - | - | Assumed cost-free as texts are already being sent | | | |
| **Childcare** |  |  |  |  | | | |
| Playgroup | £2.50 | - | - | Average childcare costs: Money Advice Service | | | |
| Childminder | £4.57 | - | - | Childcare Survey 2019 | | | |
| Family friend | £4.57 | - | - | Assumed equal to childminder cost | | | |
| Day nursery | £5.16 | - | - | Childcare Survey 2019 | | | |
| **Parenting classes** |  |  |  |  | | | |
| All parenting programmes | £1,501.00 | - | - | PSSRU Unit Costs of Health and Social Care 2016 | | | |
| **Health and social care** |  |  |  |  | | | |
| GP surgery visit | £33.00 | - | - | Personal Social Services Research Unit, 2019 | | | |
| GP home visit | £107.07 | - | - | Personal Social Services Research Unit, 2010 | | | |
| GP doctor phone call | £15.32 | - | - | Personal Social Services Research Unit, 2019 | | | |
| GP nurse surgery visit | £5.69 | - | - | Personal Social Services Research Unit, 2019 | | | |
| GP nurse home visit | £31.44 | - | - | Personal Social Services Research Unit, 2010 | | | |
| GP nurse phone call | £6.00 | - | - | Personal Social Services Research Unit, 2019 | | | |
| Health visitor | £93.35 | - | - | NHS Reference cost 2017/18 [N03B-N03D] | | | |
| District nurse | £39.42 | - | - | NHS Reference cost 2017/18 [N02AF] | | | |
| Other doctor appointment | £81.08 | - | - | NHS Reference cost 2017/18 [N29AF] | | | |
| Psychiatrist (adult) | £189.72 | - | - | NHS Reference cost 2017/18 [724 - Perinatal Psychiatry] | | | |
| Psychiatrist (child) | £283.66 | - | - | NHS Reference cost 2017/18 [MHSTOTHPLC] | | | |
| Psychologist | £142.82 | - | - | NHS Reference cost 2017/18 [713 - Psychotherapy] | | | |
| Other counsellor/therapist (adult) | £190.57 | - | - | NHS Reference cost 2017/18 [ MHSTOTHPLA] | | | |
| Other counsellor/therapist (child) | £283.66 | - | - | NHS Reference cost 2017/18 [ MHSTOTHPLC] | | | |
| Mental health nurse | £92.00 | - | - | Personal Social Services Research Unit, 2019 | | | |
| Accident and emergency visit | £112.41 | - | - | Personal Social Services Research Unit, 2010 | | | |
| Outpatient appointment | £143.73 | - | - | NHS Reference cost 2017/18 [Outpatient - General Surgery] | | | |
| Social worker (office visit) | £20.25 | - | - | Personal Social Services Research Unit, 2019 | | | |
| Social worker (home visit) | £29.25 | - | - | Personal Social Services Research Unit, 2019 | | | |
| Midwifery | £81.61 |  |  | NHS Reference cost 2017/18 [Outpatient - 560] | | | |
| 111 phone call | £13.87 | - | - | Turner J et al (2012) | | | |
| Dentist/Orthodontist | £123.38 | - | - | Personal Social Services Research Unit, 2019 | | | |
| Walk-in centre visit | £47.15 | - | - | NICE guideline 94 (2018) | | | |
| Dietician | £88.15 | - | - | NHS Reference cost 2017/18 [A03] | | | |
| General surgery | £143.74 | - | - | NHS Reference cost 2017/18 [A03] | | | |
| Ambulance | £190.53 | - | - | National Audit Office 2017 | | | |
| Orthopaedic appointments | £127.04 | - | - | NHS Reference cost 2017/18 [Outpatient -110] | | | |
| Smoking cessation support | £208.57 | - | - | NHS Reference cost 2017/18 [Day case -DZ58Z] | | | |
| Hearing Assessment | £83.77 | - | - | NHS Reference cost 2017/18 [CA37A] | | | |
| **Hospital admissions/procedures** |  |  |  |  | | | |
| Hospital day neonatal | £405.00 | - | - | NHS Reference cost 2017/18 [PB06J-PB06M] | | | |
| Hospital day paediatrician | £314.97 | - | - | NHS Reference cost 2017/18 [PX57A-PX57C] | | | |
| Birth | £2,026.30 | - | - | NHS Reference cost 2017/18 [NZ30A-C] | | | |
| C-section birth | £3,809.72 | - | - | NHS Reference cost 2017/18 [NZ50A-C] | | | |
| Miscarriage | £1,955.37 | - | - | NHS Reference cost 2017/18 [MB08A] | | | |
| Knee fracture surgery | £4,575.33 | - | - | NHS Reference cost 2017/18 [HE21B-HE21BD] | | | |
| General renal disorders | £3,919.51 | - | - | NHS Reference cost 2017/18 [LA09L] | | | |
| Cleft surgery | £607.04 | - | - | NHS Reference cost 2017/18 [CA66B] | | | |
| Heart surgery | £4,336.82 | - | - | NHS Reference cost 2017/18 [ED31A-ED31C] | | | |
| Gall bladder surgery | £3,640.81 | - | - | NHS Reference cost 2017/18 [GA10G] | | | |
| ^*^The minimum/maximum IY costs across participating sites (in cases where costs varied across sites) | | | |  |  |  |  |

**S8B Table. Cost-effectiveness scenario analysis**

|  | **Costs** | **QALYs** | **ICER** |  | **Costs** | **QALYs** | **ICER** |
| --- | --- | --- | --- | --- | --- | --- | --- |
| **Base case** | | | | **EQ5D-5L** | | | |
| Services as usual | £1,988.61 | 2.58680 |  | Services as usual | £1,988.61 | 2.64511 |  |
| Incredible Years | £2,609.46 | 2.61775 | £20,061 | Incredible Years | £2,609.46 | 2.66805 | £27,068 |
| **Controlling for baseline costs** | |  |  | **Broader perspective** |  |  |  |
| Services as usual | £1,775.42 | 2.58680 |  | Services as usual | £3,727.13 | 2.58680 |  |
| Incredible Years | £2,589.71 | 2.61775 | £26,312 | Incredible Years | £4,180.62 | 2.61775 | £14,653 |
| **12 participants per IY-I and IY-T group** | | |  | **Site 1 site costs** | |  |  |
| Services as usual | £1,988.61 | 2.58680 |  | Services as usual | £1,988.61 | 2.58680 |  |
| Incredible Years | £2,297.85 | 2.61775 | £9,992 | Incredible Years | £2,595.88 | 2.61775 | £19,622 |
| **Site 2 site costs** |  |  |  | **Site 4 site costs** |  |  |  |
| Services as usual | £1,988.61 | 2.58680 |  | Services as usual | £1,988.61 | 2.58680 |  |
| Incredible Years | £2,564.65 | 2.61775 | £18,613 | Incredible Years | £2,575.83 | 2.61775 | £18,974 |
| **Site 3 site costs** | |  |  | **Minimum site costs** |  |  |  |
| Services as usual | £1,988.61 | 2.58680 |  | Services as usual | £1,988.61 | 2.58680 |  |
| Incredible Years | £2,681.61 | 2.61775 | £22,392 | Incredible Years | £2,515.23 | 2.61775 | £17,016 |
