# Supplementary material for: A randomized controlled trial of a proportionate universal parenting program delivery model (E-SEE Steps) to enhance child social-emotional wellbeing
Source: PLoS One. 2022 Apr 4;17(4):e0265200. doi: 10.1371/journal.pone.0265200 (PMC8979462; doi:10.1371/journal.pone.0265200)
Supplement: S1 Text — (DOCX) [file pone.0265200.s011.docx]

**S1 Text. Economic sensitivity analysis**

Cost-effectiveness results were based on an adjusted analysis, controlling for a set of covariables using regression methods. All regression analyses controlled for treatment allocation, child age, adult age, child gender, highest qualification, and parental relationship status, while the QALY regression analyses also controlled for baseline EQ-5D (adults), baseline PHQ-9 and baseline ASQ:SE-2. Generalised linear models were used for the cost regression analysis with a log link and gamma distribution to allow for the skewed nature of cost data. Ordinary least square regressions were used for the QALY analysis. Missing cost and outcome data were imputed using multiple imputation by chained equations with predicted mean matching.

Scenario analyses explored the impacts of alternative costing approaches including applying the minimum, maximum and site-specific E-SEE Step programme-related costs recorded at trial sites and controlling for baseline costs. A further analysis also considered the use of the EQ-5D-5L value set.
